# Impact of funding on biomedical research: a retrospective cohort study

**DOI:** 10.1186/1471-2458-6-165

**Published:** 2006-06-22

**Authors:** Evelyne Decullier, François Chapuis

**Affiliations:** 1 Clinical Epidemiology Unit; DIM-Hospices Civils de Lyon, Lyon, France; 2 Claude Bernard University Lyon 1; RECIF- Hospices Civils de Lyon, 162 av Lacassagne, 69424 Lyon cedex 03, France

## Abstract

**Background:**

Public funding is aimed at facilitating the initiation, completion and publication of research study protocols. However, no evaluation is made to investigate the impact of grant success on the conduct of biomedical research. It is therefore of great interest to compare the fate of funded protocols versus not funded: Are they initiated? Are they completed? Did the results confirm the hypothesis? Were they published? The objective was to investigate the fate of protocols submitted for funding, whether they were funded or not.

**Methods:**

Retrospective cohort study of protocols submitted for funding to the Greater Lyon regional scientific committee in 1997. Initial characteristics of protocols (design, study size, investigator status) were abstracted from archives, and follow-up characteristics (initiation, completion and publication) from a mailed questionnaire to the principal investigators.

**Results:**

Among the 142 submitted protocols, follow-up information was available for 114 (80%). As a whole, 38% of studies were funded by the Greater Lyon research committee. The rate of initiation varied from 62% for studies with no acknowledged funding to 100% for studies with both committee and other simultaneous funding. When initiated, the rate of completion was 62% for studies with at least one funding and 40% for studies without acknowledged funding. When completed, publication was reached for 77% of studies with either committee or external funding, for 58% of studies without acknowledged funding and for 37% of studies with both committee and external funding.

**Conclusion:**

Some protocols submitted for funding were initiated and completed without any funding declared. To our understanding this mean that not all protocols submitted really needed funding and also that health care facilities are unaware that they implicitly financially support and pay for biomedical research.

## Background

Public total expenditures in biomedical research have grown in recent years[1].

In the USA, the National Institutes for Health's (NIH) total appropriation rose to $28 billion in 2004 [2]. In France, the cost of public clinical research conducted in teaching hospitals and cancer centres was estimated at 116 million euros in 2000 [3], 129 million euros in 2001 (+11% in one year) [4] and 154 million euros in 2002 (+32% in two years) [5].

Despite previous increases in public budget for clinical research, competition for obtaining funds is therefore rough since the success rates for grants remained fairly the same [2] and the competition will become even tougher in a context of public financial pressures to limit the budget for biomedical research [2,6,7].

It is expected that all research funded will lead to publication and this was explicitly mentioned in the law that settled the French National funding scheme[8]. In this perspective, a study on 1996 NIH grants found that each grant lead to 7.6 manuscripts [9]. Moreover, the NIH requested in 2005 that all publications resulting from NIH-funded research be deposited in PubMed Central [10].

The French Ministry of Health has also planned regular assessment of its national funding scheme, and four evaluations have been conducted at the national level in France (on protocols funded in 1993 [11], 1994 [12], 1995 [13], and 1996 [14]). In the USA, all protocols funded by the NIH in 1979 were evaluated to investigate publication bias, but the fate of the studies was not studied [15].

None of these evaluations investigated if the funded protocols were also submitted to complementary fundings, and if these submissions were successful.

Public funding is aimed at facilitating the initiation, completion and publication of protocols. However, both French and NIH evaluations were limited to funded research; this design hampers to investigate the impact of obtaining a funding on the conduct of biomedical research.

It is therefore of great interest to compare the fate (initiation, completion and publication) of funded protocols versus not funded, as well as to compare the fate according to type of funding. Our objective was to follow all protocols submitted for funding to the regional scientific committee of Greater Lyon in 1997 and to investigate their fate according to the obtaining of funding: are these protocols funded elsewhere? Are they initiated? Are they completed? Did the results confirm the hypothesis? Do investigators consider the study results as important? Were the results published? Our hypothesis was that absence of acknowledged funding does not hamper implementation nor publication.

## Methods

All biomedical research protocols submitted for funding to a French regional scientific research committee (Greater Lyon, France) in 1997 were included.

### Data collection

All protocols submitted are archived by the regional scientific committee. Data on initial characteristics (scope, duration, and design), funding results (yes, no) and amount (converted into 1997 euros) were collected by the same person (ED) to ensure homogeneity. To confirm data extraction, main data of interest were reviewed by FC. When variables were ambiguous or not clearly stated in protocols, data were revised by both FC and ED to reach an agreement. To obtain follow-up characteristics (initiation, completion, publication), a mailed questionnaire was sent to the principal investigator of each submitted protocol. The principal investigators were contacted up to three times to obtain the completed questionnaire in 2003.

### Definitions

*Committee funding*: two kinds of regional scientific committee fundings were considered:

- National funding scheme for clinical research: In 1992, the French Ministry of Health launched a national funding scheme aiming to support biomedical research in French hospitals [8].

- Lyon hospital system's internal funding schemes include special funding for research assistants, young researchers, registered nurses, support for clinical research protected time and support for clinical research facilities.

*External funding*: investigators had to declare if the protocol had been submitted to other funding schemes, and if so, if they succeeded.

To avoid confusion between different sources of funding, protocols funded after evaluation by the regional scientific committee were labelled "committee-funded protocols" (and "not committee-funded protocols") and protocols funded after other applications were labelled "externally funded protocols" (and "not externally funded").

*Legal and methodological *definitions used are provided in table 1[16,17].

*Fate: *specific endpoints of a research protocol (initiation, completion and publication).

*Rating of study results: *Investigators were asked to self-rate the importance of their study (Table 1).

*The direction of results *was categorized as follows: protocol results were considered as "important" if the rating of the importance of the study results by the investigator was greater than 5/10 (5 being the mean on a 1 to 10 scale), and results were considered as "unimportant" when this rating was lower or equal to 5.

Scientific publication, oral presentation and grey literature [18] were based on investigators' declaration.

Publication bias is defined as the tendency on the parts of investigators, editors, and others to favour publication of research with positive results compared to research with null or negative results [19]. As a parallel, oral presentation bias is the tendency, on the parts of researchers as well as reviewers of scientific boards of meetings, to submit or accept significant results for oral presentation.

### Ethical considerations

This study was conducted according to the French law on epidemiologic and descriptive studies [16]. Data were collected anonymously on each investigator and no consent was required since no individual information was retrieved.

### Statistics

We obtained frequency distributions for all variables (means and percentages). When assessment of association was needed, χ^2 ^tests were used for qualitative variables.

We restricted analysis of publication to the cohort of completed studies. We excluded studies from the analysis when the rating of results was not provided by the investigator. To obtain an odds ratio for publication bias and oral presentation bias, we introduced these variables in a univariate logistic regression model [20].

SAS software was used for all analyses. We considered associations to be statistically significant when p values were less than 0.05.

## Results

During the year 1997, 144 protocols were submitted for funding to the Greater Lyon regional scientific committee: 63 (44%) to the national funding scheme and 81 (56%) to the Lyon Hospital system's internal funding schemes. Two protocols were only "intention letter" with no complete protocol available and were therefore not included.

A questionnaire was sent to the principal investigator of the 142 protocols included and an answer was received for 114 (80%): five investigators were not located, 18 did not answer and five others answered that they did not wish to complete the questionnaire. The association between funding status and response from the investigator was tested and did not reveal any response bias.

### Initial characteristics of the 142 protocols

Initial files were missing for five (4%) protocols. Half of the protocols involved human beings and had therefore to be evaluated by a research ethics committee as well.

(Table 2). The design was randomised controlled for 20 (14%).

Most protocols were conducted in France only (*n *= 128; 90%) and one-half of them in a single centre.

The expected number of patients was provided for 100 (74%) protocols (minimum: 5 patients; maximum: 80,000; median: 100), not applicable for 21 (14%), no estimations were calculated for 16 (12%), and were missing for five (4%).

An expected duration in months was provided for 96 (68%) protocols (minimum: 6 months, maximum: 84 and median: 24).

### Funding

Among the 142 protocols included, 54 were committee-funded (38%): 26 (42%) were submitted to the national funding scheme and 28 (35%) for HCL-internal applications. In 1997, the amount of funding after evaluation by the Lyon regional scientific committee was 3,139,000 euros (86% of which came from the national funding scheme). The funding granted ranged from 3,050 euros for a diagnostic strategy protocol to 274,400 euros for two regional studies, with a median of 34,200 euros.

### Other funding applications

In the subset of protocols for which the investigator responded to the questionnaire (114/142), 46 (40%) were committee-funded.

Simultaneous submissions for other grants or funding were declared by 38 investigators (33%; 14/46 committee-funded protocols and 24/68 that were not committee-funded). At least two other submissions for funding were declared for 34% (13/38) and at least three for 13% (5/38). These submissions were addressed to private grant applications in 18% of cases (7/38), to other public schemes (66%; 25/38) including specific associations such as cancer associations. Another 11% (4/38) declared that they decided to use previous public funding already available from another study and 5% (2/38) did not provide any answer.

**Figure 1:**
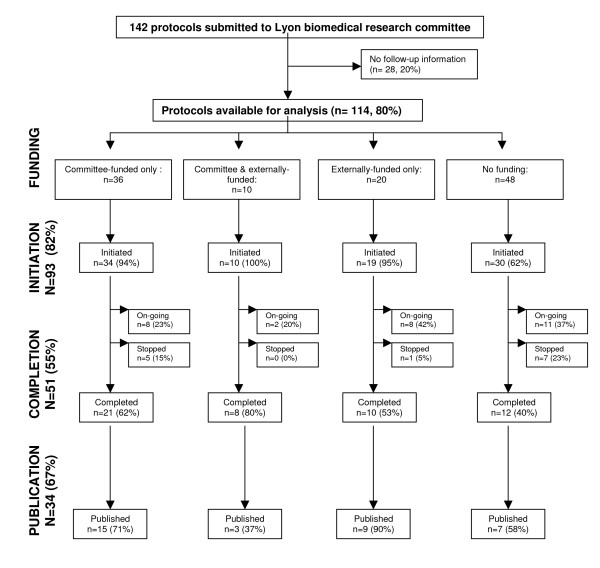
Fate of protocols submitted to a regional scientific committee.

Overall, simultaneous submissions for external funding were successful for 30 (79%), and for 6 out of 7 in case of submission to private companies. Only ten (9%) investigators declared that they obtained both committee-funding and external-funding (Figure 1).

Among the 6 private fundings, 4 did not obtained committee-funding. Considering all funding sources globally, 39% of investigators considered that all their study expenses were covered (*n *= 45), 26% that expenses were only partially covered (*n *= 30) and 10% declared that the funding was not sufficient at all (*n *= 11). Another 28 did not answer this question (25%).

### Fate

The fate of submitted protocols according to funding status is presented on Figure 1. Among the 114 answers received from investigators of submitted protocols, 93 (82%) of the studies were initiated. A total of 30 (62%) studies that were not funded were actually initiated. When both committee funding and external funding were obtained, 100% of the studies were initiated (p < 0.001). When there was at least one private funding source involved, the initiation rate was 86% and 81% otherwise (NS). On the other hand, the main reason provided by investigators to explain non-initiation of 21 protocols was absence of funding (n = 17; 81%).

Among the initiated studies, completion was declared for 8/10 (80%) committee-funded and externally funded studies, and for 12/30 (40%) studies with no funding. When there was at least one private funding source involved, the completion rate was 67% and 54% otherwise (NS). Most studies were stopped due to subject recruitment difficulties (31%), technical (methodology, time, etc) difficulties (31%) or funding difficulties (15%).

Publication rates varied from 37% for studies with both committee funding and external funding to 90% for studies with external funding only (*p *= 0.11).

When there was at least one private funding source involved the publication rate was 75% (3/4) and 66% (31/47) otherwise (NS).

#### Randomised controlled trials fate

An answer from investigator was obtained for 75% (15/20) of randomised clinical trials. The initiation and the completion rates were respectively 93% and 100% but a publication was made only for 33% (n = 5).

### Rating of study results

Investigators of initiated studies were asked to rate the importance of their study results. An answer was obtained for 74 of the 93 initiated studies. The study was still on-going for half of the non-responses. The overall mean ranking was 6.5/10 (Table 3). For the 47 completed studies, the mean rating was 6.7. The rating was greater than 5/10 ("important result") for 37 (79%).

Results were considered as being "important" for all protocols in which there was at least one private funding source involved (rating of 7/10 for the 4 protocols),

### Publication bias

For publication bias analysis on 51 completed studies, we had to exclude four studies for which the rating of study results was not available (Table 4). Among the 47 remaining protocols, 17 (36%) had not been published. The main reason explaining non-publication at the time of our study was that the paper was currently being prepared, written or submitted (*n *= 11; 65%). Two investigators declared that they had no time to write (12%), one that the results were not interesting enough (6%) and three did not provide an explanation (17%).

The odds ratio for publication of important results versus unimportant results was 1.58 (95% CI: 0.37–6.71).

### Oral presentation bias

Oral presentations were made for 39 (83%) of the 47 documented and completed studies. When results were deemed unimportant, 2/10 (20%) resulted in neither publication nor oral presentation (Table 4). Moreover, externally funded protocols had the lowest rate of oral presentation only and the highest rate for publication only. The odds ratio for oral presentation of important versus unimportant results was 1.29 (95% CI: 0.22–7.65).

### Dissemination of results

Fifty-one studies were completed (whether or not they were rated by the investigators) and 34 lead to scientific publication of which 50% resulted in multiple publications. Among the 17 studies not leading to scientific publication, there was no dissemination at all for four, and grey literature was produced for the remaining 13 studies. Grey literature comprised 46% abstracts only (*n *= 6), 15% internal reports only (*n *= 2), 15% an internal report and an abstract (*n *= 2), 15% an academic doctoral thesis only (*n *= 2) and 8% a doctoral thesis, an internal report and an abstract (*n *= 1).

## Discussion

Our hypothesis was that absence of acknowledged funding does not hamper implementation nor publication. Overall, 82% of protocols submitted for funding to the Greater Lyon regional scientific committee and for which an answer of the investigator was obtained were initiated, half of these initiated studies were completed 6 years later. Even when not funded, 30 studies out of 48 were initiated (62%), confirming our main hypothesis. Moreover, we highlighted a tendency for publication bias as well as oral presentation bias, although this was not statistically significant for study power reason.

Our study was the first to include protocols submitted on a comprehensive 1-year period to different types of public funding and was also the first to provide a follow-up of protocols that were not funded, whether they obtained other grants (e.g. private funding) or were conducted without any funding at all. It was based on only one regional scientific committee. However, this is the only regional committee not specialized and in charge of all health care facilities from general practice to highly specialised hospitals, i.e., cancer facilities, psychiatry and high-level tertiary teaching hospitals. The year 1997 was chosen to ensure enough time to conduct and complete a study and to publish the results

Some variables definitions, such as study design, were a source of problems when we extracted data from the protocols. This was partly due to imprecise definitions generally used in the literature, but also to the sometimes poor quality of submitted protocols. The variable named "*p*-value of the statistical test" was not used in the analysis since it was insufficiently documented by the investigators: in some cases, no statistical test could be performed, e.g. for descriptive non-comparative designs, but in other cases the data were truly missing. For these reasons, we relied on study results rated by the investigator over 5 (on a scale of 10). This threshold was chosen in order to ensure the minimal positive feeling of the investigator about his own study.

Self-rating by investigators is a subjective indicator, but it is very relevant since the choice to publish results is not always based on statistical significance but also on a subjective perception of the importance of the study results.

The only article published on this topic provided information on protocols funded by the NIH [15]. The results of a follow-up of NIH-funded protocols were presented, but only funded and implemented protocols were included, we believe our inclusion criteria are closer to the reality as well as to the questioning of a scientific committee member. Other scientific articles, including some meta-analyses, although not dedicated to the subject, may also give some information on protocols' fate.

Reports on the French national funding scheme have also been made public but were not published and restricted to funded protocols and to only one source of public funding [11-14].

Our completion rate was 55% at 5 years, to be compared with 68% nine years later for the NIH funded protocols and at least 80% for the four French previously mentioned unpublished reports [11-14]. However in our study there were more still on-going protocols than in these reports (23% vs. 11%).

Publication rates [15,21-25], seem to differ dramatically between authors. This difference might be due to the composition of the protocol cohorts. In our literature analysis, we extracted from each paper the publication rate and the percentage of protocols funded by pharmaceutical firms. In these papers, publication rates never reach 100% but differ according to the status of the funder: when the percentage of protocols funded by pharmaceutical firms was greater than 60%, the publication rate was less than 55%. Otherwise, the publication rate was greater than 65%.

In our study, publication rate was higher for private fundings, which is not consistent with research on this subject [15,21-26]. This could be explained by the fact that protocols submitted for fundings from both public and private sectors are different from those which only targeted private sector. Our study was not designed to answer this question, moreover the small number of completed protocols with private fundings did not allow to see if this research was more likely to produce significant results as shown in other papers[26,27].

Most funded protocols were descriptive, reflecting the general situation in the 1990s when public funding was mostly aimed at the description of clinical or epidemiological situations. Today, one could find more funding for interventional/experimental protocols, and this could be checked in few years when the current ongoing protocols will have been completed and published.

Since many protocols submitted for funding were initiated without any funding declared, does this mean that not all protocols submitted really needed funding? Or does this mean that health care facilities are unaware that they financially support and pay for biomedical research? To our understanding, both situations occur.

## Conclusion

More attention should be given to the follow-up of protocols submitted to grant application, this could be eased by the creation of local/national registries. Such registries could also help to describe the total number of applications and the total amount of money allocated per protocol, and should also be used to study the follow-up and the fate of submitted protocols, whether they are funded or not.

## Competing interests

The author(s) declare that they have no competing interest.

## Authors' contributions

ED coordinated the study, managed the data, did the statistical analysis, and drafted the manuscript.

FC designed, submitted, and coordinated the study, interpreted data, and helped to draft the manuscript.

All authors read and approved the final manuscript.

## Pre-publication history

The pre-publication history for this paper can be accessed here:



## Figures and Tables

**Table 1:** Definitions

**Legal definitions**	
Huriet-Serusclat Act	French law [16] passed in 1988 and modified in 2004, providing a framework for biomedical research protocols involving human beings. This law set up a national system of research ethics committees. Every protocol involving humans in France must be approved by one of the French research ethics committees.
Intervention requiring ethics committee approval	according to the French law [16] and to the International Committee for Harmonization- Good Clinical Practices guidelines [17], each intervention made on a human subject requires prior approval of a research ethics committee. This approval is based on the evaluation of protocols, data forms, informed consent and administrative requirements.

**Methodological definitions**	

Study design	Clinical •Descriptive: description of a cohort or a case series with no intervention •Experimental: with an intervention on humans and with a control group •Analytical: a hypothesis is tested on a cohort or in a case-control study Not clinical: protocol for which the subject of research is not a human being (such as protocol on tumour characterisation, protocols on molecules, proteins, etc.).
Study initiation and completion	Investigators were asked to classify their study as initiated/not initiated and as completed/not completed and the reason for non-completion (on-going/stopped)
Rating of study results	Investigators were asked to rate the importance of their study results from 1 to 10, 1 being "not important", 10 being "very important".
Scientific publication	Investigators had to declare if study results were published or not published as a scientific paper
Oral presentation	Investigators had to declare if study results were presented orally or not (with no details on peer-review, or scope of the meeting: regional, national or international)
Grey literature	Literature not generally accessible through libraries (internal reports, thesis, abstracts, posters)

**Table 2:** Initial characteristics of the 142 protocols

		n	%
**Field of investigation:**	Epidemiology	27	19
	Diagnostic acts	23	16
	Laboratory-based	17	12
	Physiology	14	10
	Drug evaluation	12	8
	Genetics	11	8
	Psychology-sociology	6	4
	Radiotherapy	5	4
	Economic evaluations	3	2
	Sport medicine	2	1
	Surgical strategies	2	1
	Quality of care	2	1
	Others	13	9

**Investigator status:**	Hospital consultant	65	46
	Professor/hospital consultant	54	38
	Reader/hospital consultant	12	8
	Other	6	4

**Study design**	Descriptive	45	32
	Experimental	35	25
	Analytical	27	19
	Not clinical	28	20
	Not available	2	1

**Intervention requiring ethics committee approval**	Yes	59	42
	No	75	53
	Not available	3	2

**Place of research**	Hospital	88	62
	Laboratory	19	13
	Multiple	25	18
	Other places	5	4

**Centres**	Single centre	70	51
	Multicentric-national	58	42
	Multicentric-international	7	5
	Not available	2	1

Overall	Total	137	96
	Missing files	5	4

	**Total**	**142**	**100**

**Table 3:** Investigator rating of study importance according to protocol status (on a 1 to 10 scale)

**Protocol status**	***n***	**Median**	**Minimum**	**Maximum**	**Mean**
Stopped	9	5.0	1.0	7.5	4.4
On-going	18	7.0	3.0	10.0	7.0
Completed	47	7.0	3.0	9.0	6.7

**Overall**	**74**	**7.0**	**1.0**	**10.0**	**6.5**

**Table 4:** Rating of study results and funding status according to oral presentation and scientific publication

	**Overall**	**Results**		**Funding status**			
			
		Positive (rating > 5)*	Negative (rating ≤ 5)*	None	Committee-funded only	Externally-funded only	Both
	n (%)	n (%)	n (%)	n (%)	n (%)	n (%)	n (%)
							
Published only	4 (8)	4 (11)	0 (0)	2 (17)	0 (0)	2 (20)	0 (0)
Orally presented and published	28 (59)	22 (60)	6 (60)	5 (42)	13 (72)	7 (70)	3 (43)
Orally presented only	11 (23)	9 (24)	2 (20)	2 (17)	4 (22)	1 (10)	4 (57)
Not published, not orally presented	4 (8)	2 (5)	2 (20)	3 (25)	1 (6)	0 (0)	0 (0)
	47 (100)	37 (100)	10 (100)	12 (100)	18 (100)	10 (100)	7 (100)

* Investigators were asked to rate the importance of their study results from 1 to 10, 1 being "not important", 10 being "very important".
